# *N*-(3-Bromo­phen­yl)acetamide

**DOI:** 10.1107/S1600536809013294

**Published:** 2009-04-18

**Authors:** B. Thimme Gowda, Sabine Foro, Hiromitsu Terao, Hartmut Fuess

**Affiliations:** a Department of Chemistry, Mangalore University, Mangalagangotri 574 199, Mangalore, India; b Institute of Materials Science, Darmstadt University of Technology, Petersenstrasse 23, D-64287 Darmstadt, Germany; c Faculty of Integrated Arts and Sciences, Tokushima University, Minamijosanjima-cho, Tokushima 770-8502, Japan

## Abstract

The conformation of the N—H bond in the structure of the title compound, C_8_H_8_BrNO, is *anti* to the C=O bond and to the *meta*-bromo substituent of the aromatic ring in both independent mol­ecules comprising the asymmetric unit. Mol­ecules are linked through N—H⋯O hydrogen bonding into supra­molecular chains with a twisted topology.

## Related literature

For the preparation of the compound, see: Gowda *et al.* (2006 ▶). For related structures, see: Gowda *et al.* (2007 ▶, 2008 ▶, 2009 ▶).
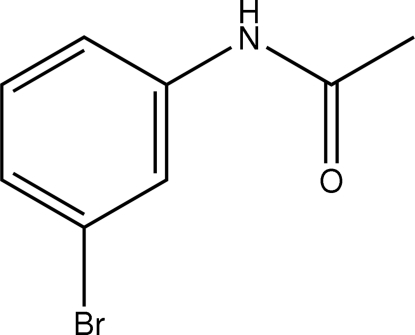


## Experimental

### 

#### Crystal data

C_8_H_8_BrNO
*M* *_r_* = 214.06
Orthorhombic, 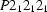

*a* = 4.7836 (6) Å
*b* = 18.765 (1) Å
*c* = 19.379 (2) Å
*V* = 1739.5 (3) Å^3^
*Z* = 8
Mo *K*α radiation
μ = 4.67 mm^−1^
*T* = 299 K
0.44 × 0.10 × 0.08 mm

#### Data collection

Oxford Diffraction Xcalibur diffractometer with a Sapphire CCD detector
Absorption correction: multi-scan (*CrysAlis RED*; Oxford Diffraction, 2007 ▶) *T*_min_ = 0.226, *T*_max_ = 0.685
9612 measured reflections
3449 independent reflections
2043 reflections with *I* > 2σ(*I*)
*R*_int_ = 0.034

#### Refinement

*R*[*F* ^2^ > 2σ(*F* ^2^)] = 0.045
*wR*(*F* ^2^) = 0.093
*S* = 0.99
3449 reflections
201 parameters
H-atom parameters constrained
Δρ_max_ = 0.31 e Å^−3^
Δρ_min_ = −0.49 e Å^−3^
Absolute structure: Flack (1983 ▶), 1366 Friedel pairs
Flack parameter: −0.008 (13)

### 

Data collection: *CrysAlis CCD* (Oxford Diffraction, 2004 ▶); cell refinement: *CrysAlis RED* (Oxford Diffraction, 2007 ▶); data reduction: *CrysAlis RED*; program(s) used to solve structure: *SHELXS97* (Sheldrick, 2008 ▶); program(s) used to refine structure: *SHELXL97* (Sheldrick, 2008 ▶); molecular graphics: *PLATON* (Spek, 2009 ▶); software used to prepare material for publication: *SHELXL97*.

## Supplementary Material

Crystal structure: contains datablocks I, global. DOI: 10.1107/S1600536809013294/tk2417sup1.cif

Structure factors: contains datablocks I. DOI: 10.1107/S1600536809013294/tk2417Isup2.hkl

Additional supplementary materials:  crystallographic information; 3D view; checkCIF report

## Figures and Tables

**Table 1:** Hydrogen-bond geometry (Å, °)

*D*—H⋯*A*	*D*—H	H⋯*A*	*D*⋯*A*	*D*—H⋯*A*
N1—H1*N*⋯O2^i^	0.86	2.05	2.887 (5)	166
N2—H2*N*⋯O1	0.86	2.10	2.953 (5)	169

Symmetry code: (i) 
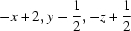
.

**Fig. 1.**
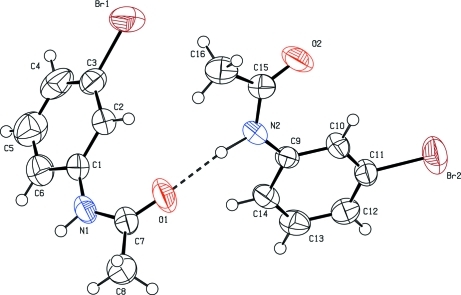
Molecular structure of (I), showing the atom labeling scheme and displacement ellipsoids drawn at the 50% probability level. The intermolecular N-H···O hydrogen is shown as a dashed line.

**Fig. 2.**
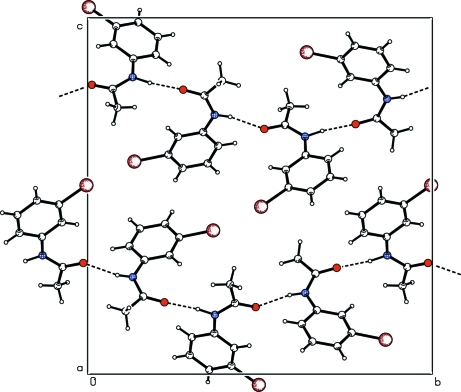
Molecular packing of (I) viewed in projection down the a-axis highlighting the supramolecular chains mediated by hydrogen bonding (shown as dashed lines).

## supplementary crystallographic information

### Comment

As part of a study of the effect of ring and side-chain substitutions on the crystal structures of aromatic amides (Gowda *et al.*, 2007, 2008, 2009), in the present work, the structure of *N*-(3-bromophenyl)acetamide (I) has been determined. The conformation of the N—H bond in the structure is *anti* to the *meta*-bromo substituent of the aromatic ring (Fig. 1), in both independent molecules comprising the asymmetric unit, similar to that observed in *N*-(3-chlorophenyl)acetamide (Gowda *et al.*, 2008). Further, the conformation of the C=O bond is *anti* to the N—H bond. The two independent molecules in (I) are linked through intermolecular N—H···O hydrogen bonding into a supramolecular chains with a twisted topology (Table 1, Fig. 2).

### Experimental

Compound (I) was prepared according to the literature method (Gowda *et al.*, 2006). Single crystals were obtained from an ethanolic solution of (I).

### Refinement

The H atoms were positioned with idealized geometry using a riding model with C—H = 0.93–0.96 Å and N—H = 0.86 Å, and with U_iso_ set to 1.2 times *U*_eq_(parent atom).

### Crystal data

**Table:**

C_8_H_8_BrNO	*F*(000) = 848
*M**_r_* = 214.06	*D*_x_ = 1.635 Mg m^−^^3^
Orthorhombic, *P*2_1_2_1_2_1_	Mo *K*α radiation, λ = 0.71073 Å
Hall symbol: P 2ac 2ab	Cell parameters from 3601 reflections
*a* = 4.7836 (6) Å	θ = 2.4–27.6°
*b* = 18.765 (1) Å	µ = 4.67 mm^−^^1^
*c* = 19.379 (2) Å	*T* = 299 K
*V* = 1739.5 (3) Å^3^	Long needle, colourless
*Z* = 8	0.44 × 0.10 × 0.08 mm

### Data collection

**Table:**

Oxford Diffraction Xcalibur diffractometer with a Sapphire CCD detector	3449 independent reflections
Radiation source: fine-focus sealed tube	2043 reflections with *I* > 2σ(*I*)
graphite	*R*_int_ = 0.034
Rotation method data acquisition using ω and φ scans	θ_max_ = 26.4°, θ_min_ = 2.4°
Absorption correction: multi-scan (*CrysAlis RED*; Oxford Diffraction, 2007)	*h* = −5→5
*T*_min_ = 0.226, *T*_max_ = 0.685	*k* = −22→23
9612 measured reflections	*l* = −20→24

### Refinement

**Table:**

Refinement on *F*^2^	Secondary atom site location: difference Fourier map
Least-squares matrix: full	Hydrogen site location: inferred from neighbouring sites
*R*[*F*^2^ > 2σ(*F*^2^)] = 0.045	H-atom parameters constrained
*wR*(*F*^2^) = 0.093	*w* = 1/[σ^2^(*F*_o_^2^) + (0.0455*P*)^2^ + 0.0252*P*] where *P* = (*F*_o_^2^ + 2*F*_c_^2^)/3
*S* = 0.99	(Δ/σ)_max_ = 0.001
3449 reflections	Δρ_max_ = 0.31 e Å^−^^3^
201 parameters	Δρ_min_ = −0.49 e Å^−^^3^
0 restraints	Absolute structure: Flack (1983), 1366 Friedel pairs
Primary atom site location: structure-invariant direct methods	Flack parameter: −0.008 (13)

### Special details

**Table:**

Experimental. Absorption correction: CrysAlis RED, Oxford Diffraction Ltd. (2007). Empirical absorption correction using spherical harmonics, implemented in SCALE3 ABSPACK scaling algorithm.
Geometry. All e.s.d.'s (except the e.s.d. in the dihedral angle between two l.s. planes) are estimated using the full covariance matrix. The cell e.s.d.'s are taken into account individually in the estimation of e.s.d.'s in distances, angles and torsion angles; correlations between e.s.d.'s in cell parameters are only used when they are defined by crystal symmetry. An approximate (isotropic) treatment of cell e.s.d.'s is used for estimating e.s.d.'s involving l.s. planes.
Refinement. Refinement of *F*^2^ against ALL reflections. The weighted *R*-factor *wR* and goodness of fit *S* are based on *F*^2^, conventional *R*-factors *R* are based on *F*, with *F* set to zero for negative *F*^2^. The threshold expression of *F*^2^ > σ(*F*^2^) is used only for calculating *R*-factors(gt) *etc*. and is not relevant to the choice of reflections for refinement. *R*-factors based on *F*^2^ are statistically about twice as large as those based on *F*, and *R*- factors based on ALL data will be even larger.

### Fractional atomic coordinates and isotropic or equivalent isotropic displacement parameters (Å^2^)

**Table:**

	*x*	*y*	*z*	*U*_iso_*/*U*_eq_	
Br1	0.64658 (17)	0.36438 (3)	0.40336 (3)	0.0961 (3)	
O1	1.0611 (8)	0.22271 (18)	0.20168 (19)	0.0786 (12)	
N1	0.9531 (7)	0.13129 (19)	0.27225 (19)	0.0531 (10)	
H1N	0.9782	0.0863	0.2779	0.064*	
C1	0.7804 (10)	0.1647 (2)	0.3206 (2)	0.0492 (12)	
C2	0.7903 (10)	0.2377 (2)	0.3338 (2)	0.0524 (12)	
H2	0.9111	0.2670	0.3092	0.063*	
C3	0.6192 (12)	0.2656 (3)	0.3838 (2)	0.0606 (14)	
C4	0.4398 (12)	0.2247 (4)	0.4216 (3)	0.0740 (17)	
H4	0.3269	0.2450	0.4553	0.089*	
C5	0.4300 (12)	0.1519 (3)	0.4084 (3)	0.0848 (18)	
H5	0.3068	0.1234	0.4331	0.102*	
C6	0.6000 (11)	0.1215 (3)	0.3592 (3)	0.0691 (15)	
H6	0.5951	0.0726	0.3516	0.083*	
C7	1.0850 (10)	0.1608 (3)	0.2180 (2)	0.0546 (13)	
C8	1.2664 (11)	0.1102 (2)	0.1767 (2)	0.0726 (17)	
H8A	1.2240	0.0620	0.1895	0.087*	
H8B	1.4597	0.1199	0.1861	0.087*	
H8C	1.2303	0.1166	0.1284	0.087*	
Br2	0.30944 (16)	0.49664 (3)	−0.03009 (3)	0.0919 (3)	
O2	0.9050 (8)	0.48750 (17)	0.18809 (18)	0.0709 (10)	
N2	0.8933 (8)	0.36972 (18)	0.16644 (18)	0.0475 (9)	
H2N	0.9634	0.3294	0.1785	0.057*	
C9	0.6865 (9)	0.3675 (2)	0.11456 (19)	0.0397 (10)	
C10	0.6162 (10)	0.4252 (2)	0.0737 (2)	0.0464 (11)	
H10	0.7039	0.4690	0.0799	0.056*	
C11	0.4147 (10)	0.4168 (2)	0.0239 (2)	0.0499 (12)	
C12	0.2819 (9)	0.3533 (3)	0.0127 (2)	0.0528 (12)	
H12	0.1476	0.3489	−0.0218	0.063*	
C13	0.3519 (12)	0.2959 (3)	0.0536 (2)	0.0576 (13)	
H13	0.2619	0.2525	0.0473	0.069*	
C14	0.5532 (9)	0.3025 (2)	0.1035 (2)	0.0490 (12)	
H14	0.6013	0.2632	0.1302	0.059*	
C15	0.9939 (9)	0.4277 (3)	0.1993 (2)	0.0490 (12)	
C16	1.2135 (10)	0.4133 (3)	0.2521 (3)	0.0652 (14)	
H16A	1.2663	0.3640	0.2502	0.078*	
H16B	1.1416	0.4242	0.2971	0.078*	
H16C	1.3739	0.4426	0.2429	0.078*	

### Atomic displacement parameters (Å^2^)

**Table:**

	*U*^11^	*U*^22^	*U*^33^	*U*^12^	*U*^13^	*U*^23^
Br1	0.1400 (6)	0.0715 (4)	0.0768 (4)	0.0319 (4)	−0.0121 (4)	−0.0259 (3)
O1	0.110 (3)	0.046 (2)	0.080 (2)	0.013 (2)	0.022 (2)	0.0251 (19)
N1	0.067 (3)	0.035 (2)	0.057 (2)	0.0055 (19)	0.003 (2)	0.012 (2)
C1	0.058 (3)	0.045 (3)	0.044 (3)	0.005 (2)	−0.005 (2)	0.006 (2)
C2	0.065 (3)	0.045 (3)	0.047 (2)	0.004 (2)	−0.003 (3)	0.003 (2)
C3	0.075 (4)	0.064 (3)	0.043 (3)	0.021 (3)	−0.007 (3)	−0.006 (3)
C4	0.068 (4)	0.108 (5)	0.046 (3)	0.021 (3)	0.008 (3)	−0.009 (3)
C5	0.089 (5)	0.096 (5)	0.070 (4)	−0.020 (3)	0.014 (4)	0.002 (4)
C6	0.083 (4)	0.059 (3)	0.065 (3)	−0.009 (3)	0.003 (3)	0.009 (3)
C7	0.067 (4)	0.045 (3)	0.052 (3)	0.007 (2)	0.002 (3)	0.008 (2)
C8	0.086 (5)	0.065 (3)	0.066 (3)	0.005 (3)	0.012 (3)	0.002 (3)
Br2	0.1222 (5)	0.0670 (4)	0.0866 (4)	0.0074 (4)	−0.0273 (4)	0.0277 (3)
O2	0.091 (3)	0.0351 (18)	0.086 (2)	0.0039 (19)	−0.014 (2)	−0.0108 (17)
N2	0.058 (3)	0.0299 (19)	0.054 (2)	0.0034 (19)	0.000 (2)	0.0001 (18)
C9	0.044 (3)	0.035 (2)	0.040 (2)	0.000 (2)	0.004 (2)	−0.003 (2)
C10	0.051 (3)	0.035 (2)	0.053 (3)	−0.002 (2)	−0.001 (3)	0.005 (2)
C11	0.059 (3)	0.044 (3)	0.047 (3)	0.008 (2)	0.003 (3)	0.007 (2)
C12	0.048 (3)	0.058 (3)	0.052 (3)	−0.002 (2)	−0.003 (2)	−0.006 (2)
C13	0.056 (3)	0.051 (3)	0.066 (3)	−0.002 (3)	−0.007 (3)	−0.011 (3)
C14	0.053 (3)	0.035 (3)	0.058 (3)	0.000 (2)	0.006 (3)	−0.002 (2)
C15	0.050 (3)	0.047 (3)	0.050 (3)	−0.001 (3)	−0.001 (3)	−0.007 (3)
C16	0.065 (4)	0.062 (3)	0.069 (3)	−0.009 (3)	−0.007 (3)	−0.012 (3)

### Geometric parameters (Å, °)

**Table:**

Br1—C3	1.897 (5)	Br2—C11	1.895 (4)
O1—C7	1.209 (5)	O2—C15	1.220 (5)
N1—C7	1.346 (5)	N2—C15	1.349 (5)
N1—C1	1.398 (5)	N2—C9	1.411 (5)
N1—H1N	0.8600	N2—H2N	0.8600
C1—C2	1.394 (6)	C9—C10	1.383 (5)
C1—C6	1.400 (6)	C9—C14	1.392 (6)
C2—C3	1.372 (6)	C10—C11	1.374 (6)
C2—H2	0.9300	C10—H10	0.9300
C3—C4	1.364 (7)	C11—C12	1.369 (6)
C4—C5	1.390 (7)	C12—C13	1.377 (6)
C4—H4	0.9300	C12—H12	0.9300
C5—C6	1.377 (7)	C13—C14	1.371 (6)
C5—H5	0.9300	C13—H13	0.9300
C6—H6	0.9300	C14—H14	0.9300
C7—C8	1.514 (6)	C15—C16	1.490 (6)
C8—H8A	0.9600	C16—H16A	0.9600
C8—H8B	0.9600	C16—H16B	0.9600
C8—H8C	0.9600	C16—H16C	0.9600
			
C7—N1—C1	128.1 (4)	C15—N2—C9	127.6 (4)
C7—N1—H1N	116.0	C15—N2—H2N	116.2
C1—N1—H1N	116.0	C9—N2—H2N	116.2
C2—C1—N1	123.0 (4)	C10—C9—C14	119.1 (4)
C2—C1—C6	119.5 (5)	C10—C9—N2	123.7 (4)
N1—C1—C6	117.5 (4)	C14—C9—N2	117.2 (4)
C3—C2—C1	119.0 (5)	C11—C10—C9	118.9 (4)
C3—C2—H2	120.5	C11—C10—H10	120.5
C1—C2—H2	120.5	C9—C10—H10	120.5
C4—C3—C2	122.7 (5)	C12—C11—C10	122.5 (4)
C4—C3—Br1	119.1 (4)	C12—C11—Br2	118.5 (4)
C2—C3—Br1	118.2 (4)	C10—C11—Br2	119.0 (3)
C3—C4—C5	118.3 (5)	C11—C12—C13	118.4 (4)
C3—C4—H4	120.8	C11—C12—H12	120.8
C5—C4—H4	120.8	C13—C12—H12	120.8
C6—C5—C4	121.0 (5)	C14—C13—C12	120.4 (4)
C6—C5—H5	119.5	C14—C13—H13	119.8
C4—C5—H5	119.5	C12—C13—H13	119.8
C5—C6—C1	119.6 (5)	C13—C14—C9	120.6 (4)
C5—C6—H6	120.2	C13—C14—H14	119.7
C1—C6—H6	120.2	C9—C14—H14	119.7
O1—C7—N1	123.7 (4)	O2—C15—N2	122.2 (4)
O1—C7—C8	121.3 (4)	O2—C15—C16	122.3 (4)
N1—C7—C8	115.0 (4)	N2—C15—C16	115.5 (4)
C7—C8—H8A	109.5	C15—C16—H16A	109.5
C7—C8—H8B	109.5	C15—C16—H16B	109.5
H8A—C8—H8B	109.5	H16A—C16—H16B	109.5
C7—C8—H8C	109.5	C15—C16—H16C	109.5
H8A—C8—H8C	109.5	H16A—C16—H16C	109.5
H8B—C8—H8C	109.5	H16B—C16—H16C	109.5
			
C7—N1—C1—C2	−22.3 (7)	C15—N2—C9—C10	21.5 (6)
C7—N1—C1—C6	160.8 (4)	C15—N2—C9—C14	−160.2 (4)
N1—C1—C2—C3	−177.9 (4)	C14—C9—C10—C11	0.7 (6)
C6—C1—C2—C3	−1.0 (7)	N2—C9—C10—C11	178.9 (4)
C1—C2—C3—C4	0.4 (7)	C9—C10—C11—C12	−0.5 (7)
C1—C2—C3—Br1	177.6 (3)	C9—C10—C11—Br2	178.0 (3)
C2—C3—C4—C5	−0.4 (8)	C10—C11—C12—C13	0.7 (7)
Br1—C3—C4—C5	−177.6 (4)	Br2—C11—C12—C13	−177.8 (4)
C3—C4—C5—C6	1.0 (8)	C11—C12—C13—C14	−1.1 (7)
C4—C5—C6—C1	−1.7 (8)	C12—C13—C14—C9	1.2 (7)
C2—C1—C6—C5	1.7 (7)	C10—C9—C14—C13	−1.0 (6)
N1—C1—C6—C5	178.7 (4)	N2—C9—C14—C13	−179.4 (4)
C1—N1—C7—O1	−3.1 (8)	C9—N2—C15—O2	2.2 (7)
C1—N1—C7—C8	177.9 (4)	C9—N2—C15—C16	−179.9 (4)

### Hydrogen-bond geometry (Å, °)

**Table:**

*D*—H···*A*	*D*—H	H···*A*	*D*···*A*	*D*—H···*A*
N1—H1N···O2^i^	0.86	2.05	2.887 (5)	166
N2—H2N···O1	0.86	2.10	2.953 (5)	169

Symmetry codes: (i) −*x*+2, *y*−1/2, −*z*+1/2.
